# Olanzapine (5 mg) plus standard triple antiemetic therapy for the prevention of multiple-day cisplatin hemotherapy-induced nausea and vomiting: a prospective randomized controlled study

**DOI:** 10.1007/s00520-022-07067-6

**Published:** 2022-04-21

**Authors:** Jiali Gao, Jun Zhao, Caihong Jiang, Feng Chen, Lanzhen Zhao, Ying Jiang, Hui Li, Wenjuan Wang, Yungaowa Wu, Yilan Jin, Lenggaowa Da, Guang Liu, Yajuan Zhang, Hongxia Li, Zewei Zhang, Gaowa Jin, Quanfu Li

**Affiliations:** 1 Department of Medical Oncology, Ordos Central Hospital, Ordos, 017000 China; 2 Department of Radiation Oncology, State Key Laboratory of Oncology in South China, Collaborative Innovation Center for Cancer Medicine, Sun Yat-Sen University Cancer Center, Guangzhou, Guangdong People’s Republic of China

**Keywords:** Olanzapine, Aprepitant, CINV, Multiple-day cisplatin chemotherapy

## Abstract

**Objective:**

A prospective randomized controlled trial was conducted to compare 5 mg olanzapine plus standard triple antiemetic therapy for the prevention of nausea and vomiting induced by multiple-day cisplatin chemotherapy.

**Methods:**

Patients who received a 3-day cisplatin-based chemotherapy (25 mg/m^2^/d) were given either 5 mg olanzapine plus triple therapy with aprepitant, tropisetron, and dexamethasone (quadruple group) or 5 mg olanzapine plus tropisetron and dexamethasone, omitting aprepitant (triplet group). The primary endpoint was the complete response (CR) in the overall phase (OP) (0–120 h) between quadruple group and triplet group. The secondary endpoints were the CR in the acute phase (AP) (0–24 h) and delayed phase (DP) (25–120 h) between two groups. The first time of vomiting was also compared by Kaplan–Meier curves. The impact of chemotherapy-induced nausea and vomiting (CINV) on the quality of life was assessed by the Functional Living Index-Emesis (FLIE). Aprepitant-related adverse effects (AEs) were also recorded.

**Results:**

(1) The primary endpoint CR during OP was 76.0% (45/59) vs 67.0% (41/61) between the quadruple group and triplet group (*P* = 0.271). The secondary endpoint CR during the AP was significantly higher in the quadruple group than in the triplet group, which was 100.0% (59/59) vs 93.0% (57/61) (*P* = 0.045). The difference of CR during delayed phase between the groups was especially higher in the quadruple group compared to the triplet group (76.0% (45/59) vs 67.0% (41/61) (*P* = 0.271)). The rate of patients who achieved total protection in the overall phase was also higher in the quadruple group than the triplet group (28.8% (17/59) vs 23.0% (14/61) (*P* = 0.463)). During the OP, the incidence of no vomiting in the quadruple group and the triplet group was 93.2% (55/59) vs 80.3% (49/61) (*P* = 0.038), respectively. (2) Kaplan–Meier curves of time to first emesis were obviously longer in the quadruple group compared with the triplet group (*P* = 0.031). According to FLIE, no impact of CINV on daily life was defined as total score of questionnaire > 108; this study exhibited identical life quality between two groups. (3) The most common aprepitant- or olanzapine-related AEs included sedation, fatigue, and constipation. The occurrences between two groups were identical.

**Conclusion:**

It may been recommended that 5 mg olanzapine plus tropisetron and dexamethasone, omitting aprepitant triplet regimen as an alternative therapy in prevention CINV induced by multiple-day cisplatin chemotherapy due to the excellent CINV control rate and safety.

## Introduction

Patients receiving multiple-day cisplatin chemotherapy are at risk of both acute and delayed nausea and vomiting for each day, as acute and delayed emesis may overlap after the initial-day chemotherapy until the last day of chemotherapy[1, 2]. Although the combination of aprepitant, 5-HT3 receptor antagonist (5-HT3RA), and dexamethasone (DXM) had showed higher complete response than the combination of 5-HT3RA plus dexamethasone in cisplatin multiple-day chemotherapy clinical studies, nausea remains a major problem for many patients[3–5]. The activity of olanzapine on multiple receptors, particularly the D2, 5-HT2c, and 5-HT3 receptors, may be involved in nausea and vomiting. A single-institution phase 3 trial showed that olanzapine was comparable to aprepitant in the control of CINV, and nausea was better controlled with olanzapine in delayed period and overall period[6]. Somnolence is a major side effect when olanzapine was administered at a dose of 10 mg. In a phase 2 study, 5 mg olanzapine has shown equivalent activity to 10 mg olanzapine and a favourable safety in relation to somnolence[7]. Guidelines suggest that a dose reduction to 5 mg should be considered to prevent sedation, and a randomized, double-blind, placebo-controlled phase 3 study showed that 5 mg olanzapine combined with aprepitant, palonosetron, and dexamethasone could be a new standard antiemetic therapy for patients undergoing cisplatin single-day chemotherapy[8]. Aprepitant is an oral neurokinin-1 receptor antagonist[9]. A randomized controlled trial was conducted to compare the efficacy of 5 mg olanzapine plus triple therapy with aprepitant, tropisetron, and dexamethasone versus 5 mg olanzapine plus tropisetron and dexamethasone, omitting aprepitant in prevention of CINV in patients receiving multiple-day cisplatin chemotherapy (chiCTR1800018424).

## Methods

### Patients

This study was approved by the local Institutional Review Board, and all patients provided written informed consent before the start of study procedures. A randomized, clinical trial (chiCTR1800018424) was conducted to compare the effectiveness of 5 mg olanzapine plus triple therapy with aprepitant, tropisetron, and dexamethasone group versus 5 mg olanzapine plus tropisetron and dexamethasone group, omitting aprepitant in prevention of CINV in patients receiving multiple-day cisplatin chemotherapy.

### Endpoints

We chose the CR rate, defined as the absence of emetic episodes and no use of rescue medications during the OP after the initiation of cisplatin, as the primary endpoint. Secondary endpoints are the CR rate in the AP and the DP. The total control (TC) rate is defined as the absence of nausea and emetic episodes and no use of rescue medications during OP. We used a 100 mm categorical scale to stratify nausea and chose ≤ 5 mm to define the TC. The time of treatment failure is defined as the time of first emetic episode or the use of rescue medication. AEs were graded according to Common Terminology Criteria for Adverse Events (CTCAE) v.4.0.

### Randomization

After confirming that the patients fulfill the eligibility criteria, the patients are randomized by random digits table.

### Eligibility criteria

#### Inclusion criteria

1. Patients older than 18 years who will receive 3-day cisplatin-based chemotherapy (25 mg/m^2^/d) enroled in the study. All patients had histologically confirmed.
2. Karnofsky Performance Scale ≥ 70
3. There is no abnormality in liver and kidney function, blood routine, and electrocardiogram before chemotherapy, including white blood cell count > 3.5 × 10^9/L, absolute neutrophil count > 1.5 × 10^9/L, platelet count > 85 × 10^9/L, alkaline phosphatase < 2.5 upper limit of normal (ULN), alanine transaminase < 2.5 upper limit of normal (ULN), bilirubin < 1.5 ULN, and creatinine < 1.5 ULN.
4. Patients without chemotherapy contraindications
5. No episodes of nausea and vomiting occurred during the last 1 week before enrolment, and no aprepitant or olanzapine was used for pretreatment.
6. Patients are able to understand and describe patient-reported outcomes.

#### Exclusion criteria

Patients were excluded if they meet any of the following criteria:

1. symptomatic brain metastases;
2. requiring treatment for ascites or pleural effusion;
3. requiring radiotherapy in the abdominal or pelvic field;
4. requiring anticonvulsant medication;
5. history of hypersensitivity or allergy to the study drugs or similar compounds;
6. severe complications (pulmonary fibrosis, heart failure, myocardial infarction, unstable angina, cerebral vascular disorder, psychiatric disease, renal dysfunction, liver dysfunction, intestinal paresis, ileus, uncontrollable diabetes mellitus, active peptic ulcer, etc.);
7. history of using any of the following drugs within 48 h: opioids, aprepitant, 5-HT3 receptor antagonists, dexamethasone, dopamine receptor antagonists, antihistamines, benzodiazepines, and phenothiazine antipsychotics;
8. pregnant or lactating women or women with child-bearing potential or men wishing to be the father of children;
9. partial/complete bowel obstruction; and
10.  malignant tumor of digestive tract.

### Treatments

The study of antiemetic administrations are shown in Table 1. Patients in quadruple group received the following: aprepitant 125 mg PO day 1, 80 mg PO days 2–3 (EMEND, MSD Sharp & Dohme, Haar, Germany), olanzapine 5 mg PO days 1–3 (Jiangsu Haosen Pharmaceutical Co., Ltd.), tropisetron 5 mg iv days 1–3 (Beijing Shuanglu Pharmaceutical Co. Ltd., China), and dexamethasone 5 mg iv days 1–3. Patients in the triplet group received the following: olanzapine 5 mg PO days 1–3, tropisetron 5 mg iv days 1–3, and dexamethasone 10 mg iv days 1–3. The aprepitant group had a half dosage of dexamethasone besides tropisetron hydrochloride and aprepitant, since the function of CYP3A4 in DXM pharmacokinetics could be exhibited by aprepitant[10].

**Table 1:** Antiemetic administrations

	Day 1	Day 2	Day 3
Quadruple antiemetic regimen	Aprepitant 125 mg PO	Aprepitant 80 mg PO	Aprepitant 80 mg PO
	Olanzapine 5 mg PO	Olanzapine 5 mg PO	Olanzapine 5 mg PO
	Tropisetron 5 mg iv	Tropisetron 5 mg iv	Tropisetron 5 mg iv
	Dexamethasone 5 mg PO	Dexamethasone 5 mg PO	Dexamethasone 5 mg PO
Triple antiemetic regimen	Olanzapine 5 mg PO	Olanzapine 5 mg PO	Olanzapine 5 mg PO
	Tropisetron 5 mg iv	Tropisetron 5 mg iv	Tropisetron 5 mg iv
	Dexamethasone 10 mg PO	Dexamethasone 10 mg PO	Dexamethasone 10 mg PO

### Follow-up

Patients recorded and self-reported the times and dates of vomiting or retching episodes, and the use of rescue therapy from the time of chemotherapy infusion (0 h) until day 5. Patients were contacted in the mornings of days 2–5 to ensure compliance with nausea categorical scale. Functional Living Index-Emesis (FLIE) questionnaire scoring was self-administered early on day 5, directly following completion of final self-reports[11]. Notably, FLIE is a validated emesis- and nausea-specific questionnaire with nine nausea domain questions (items) and nine vomiting domain questions (items) and “no impact of CINV on daily life” represented mean scores > 6 on a 7-point scale (> 108 in total)[12, 13].

All patients underwent post-treatment examination on days 6–8 and follow-up at days 19–21, and AEs related to aprepitant and olanzapine were recorded.

### Statistical analysis

The sponsor managed the data and performed the analyses for this study. The hypothesis of this study was that the CR rate of 5 mg olanzapine plus triple therapy with aprepitant, tropisetron, and dexamethasone quadruple group would be significantly higher than that of 5 mg olanzapine plus tropisetron and dexamethasone group, omitting aprepitant triplet group. Other trials have shown that the CR rate of triple antiemetic therapy was about 65%[14, 15]. According to the previous studies, CR rates of antiemesis treatment by olanzapine combined with aprepitant, tropisetron, and dexamethasone were 86%[14]. We believed that an improvement of more than 15% in the CR rate would be clinically meaningful. Therefore, assuming that the null hypothesis of the CR rate is 65% and the alternative hypothesis is 80%, we calculated that a minimum of 82 patients was required to achieve a one-sided type 1 error of 0.1% and 80% of power, based on the exact binomial distribution. Because some dropouts were expected, we set the target sample size to 104, and the sample size calculation was performed by SASV.9.4 (Cary, NC, USA).

Treatment comparisons were made using logistic regression models that included terms for treatment, gender, age, alcohol use, history of motion sickness, etc. All comparisons used a two-sided significance level of 5%. Tests of significance were based on the logistic regression models, and the nominal *P-*values were reported. Kaplan–Meier curves of time to first emesis were constructed to both groups. Fisher’s exact test was used to compare the percentage of patients who got CR or experienced aprepitant-related AEs between the two groups.

## Results

### Patients

From March 2018 to March 2019, this prospective, randomized, controlled study was conducted at the Medical Oncology Department of Ordos Central Hospital in Inner Mongolia, China. A total of 132 patients are assigned to a study group with the use of random digits table. Six patients did not receive treatment (because of the cancellation of chemotherapy), and six patients dropped out of the study because of lack of nausea data and FLIE questionnaires. Thus, 59 patients in the quadruple group and 61 in the triple group were completely assessed. Among the 120 patients, all received 3-day cisplatin-based double regimens combined with one of the following chemotherapeutic drugs: gemcitabine, docetaxel, etoposide, pemetrexed, paclitaxel, capecitabine and irinotecan, sometimes plus bevacizumab or Herceptin, etc. The baseline characteristics were comparable between two groups (Table 2).

**Table 2:** Patients’ baseline characteristics (*n* [%])

Characteristics	Quadruple group (*n* = 59)	Triple group (*n* = 61)	*P*
Age (years)			
≥ 55	60.39 ± 9.22	58.11 ± 7.80	0.104
Gender			0.708
Female	27 (45.76)	30 (49.18)	
Male	32 (54.24)	31 (50.82)	
History of motion sickness	9 (15.25)	14 (22.95)	0.284
History of nausea with pregnancy in female	14 (51.85)	20 (66.67)	0.271
Alcohol use			0.881
No consumption	32 (54.24)	32 (52.46)	
< 4 drinks per week	19 (32.20)	22 (36.07)	
≥ 4 drinks per week	8 (13.56)	7 (11.48)	
Smoking index			0.144
No smoking	15 (25.42)	22 (36.07)	
0 ~ 400	10 (16.95)	4 (6.56)	
≥ 400	34 (57.63)	35 (57.38)	
Type of malignance			0.850
Lung cancer	31 (52.54)	31 (50.82)	
Others	28 (47.46)	30 (49.18)	
Chemotherapy cycle			0.109
First cycle	25 (42.37)	17 (27.87)	
Second to third cycle	22 (37.29)	22 (36.07)	
≥ fourth cycle	12 (20.34)	22 (36.07)	

### Efficacy

**Fig. 1:**
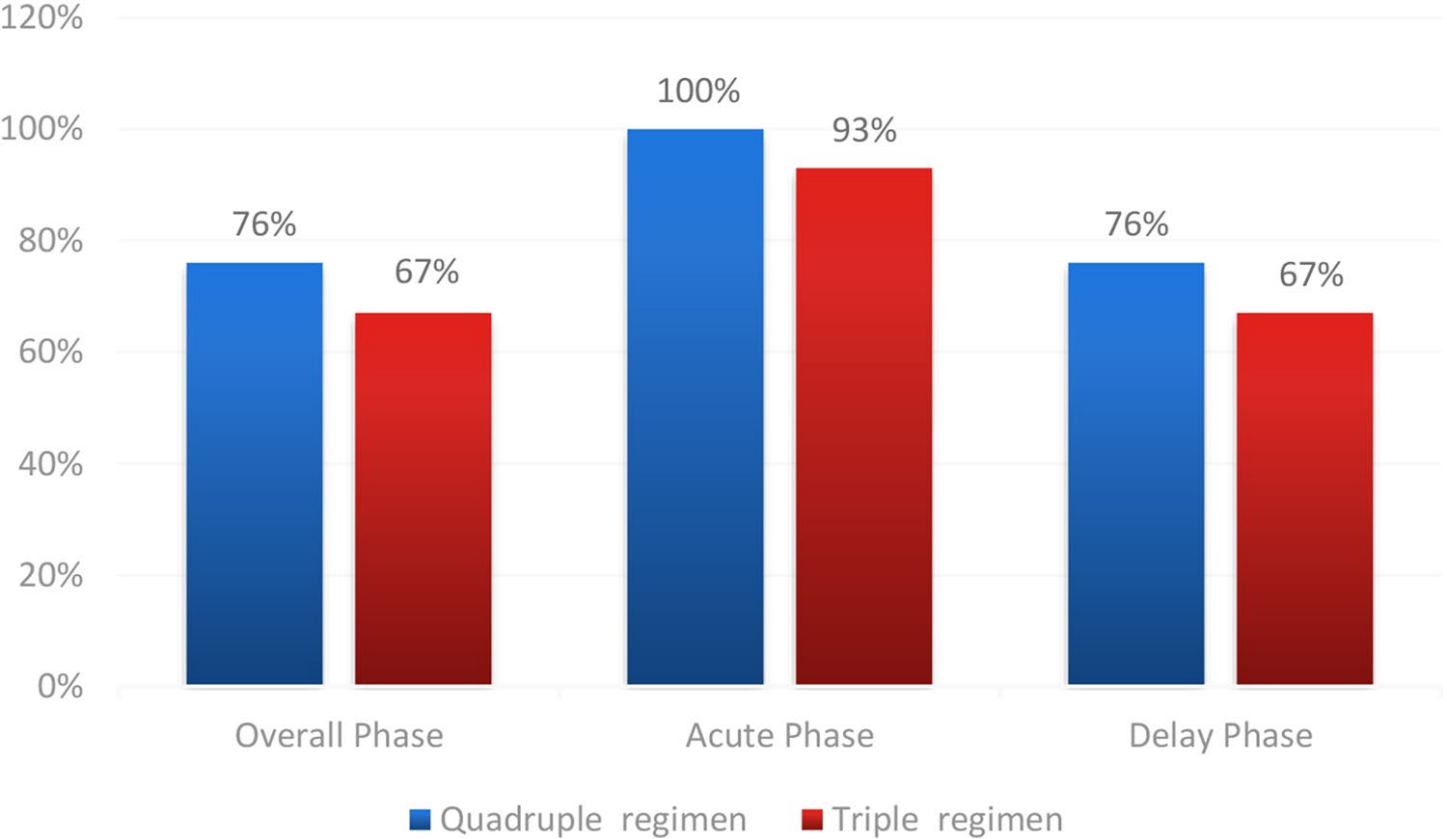
Comparison of complete response between two groups

The primary endpoint of CR rate during the overall phase in the quadruple group (76.0% (45/59)) was higher than those in the triplet group (67.0% (41/61)) (*P* = 0.271), but there was no statistical significance. During AP, the CR of quadruple group (100.0% (59/59)) was significantly higher than triplet group (93.0% (57/61)) (*P* = 0.045). The difference between the groups was especially greater in the delayed phase (24–120 h) (quadruple group 76.0% (45/59) vs triplet group 67.0% (41/61) (*P* = 0.271)) (Fig. 1). The total protection rates of quadruple group (28.8% (17/59)) in the overall phase were also larger than in the triplet group (23.0% (14/61) (*P* = 0.463)). During the OP, the incidence of no vomiting in quadruple group and triplet group was 93.2% (55/59) vs 80.3% (49/61) (*P* = 0.038), respectively. In the no-rescue treatment, few cases were reported in the quadruple group (16.9% (10/59)) than in the triplet group (27.9% (17/61) (*P* = 0.152)) during the OP.

### Comparison of FLIE index

According to FLIE, reports of no impact of CINV on daily life were exhibited by 47.5% (28/59) of the quadruple group and 44.3% (27/61) of the triplet group (*P* = 0.035). The comparison of FLIE index of nausea or vomiting between two groups was listed below in Table 3.

**Table 3:** Comparison of FLIE index

Items	Quadruple group	Triple group	*P*
Nausea FLIE score	48.92 ± 12.32	48.66 ± 12.15	0.907
Vomiting FLIE score	52.91 ± 11.49	50.67 ± 13.05	0.322
FLIE score	101.83 ± 22.46	99.33 ± 24.70	0.563

*FLIE*, Functional Living Index-Emesis

### Comparison of time to first vomiting

**Fig. 2:**
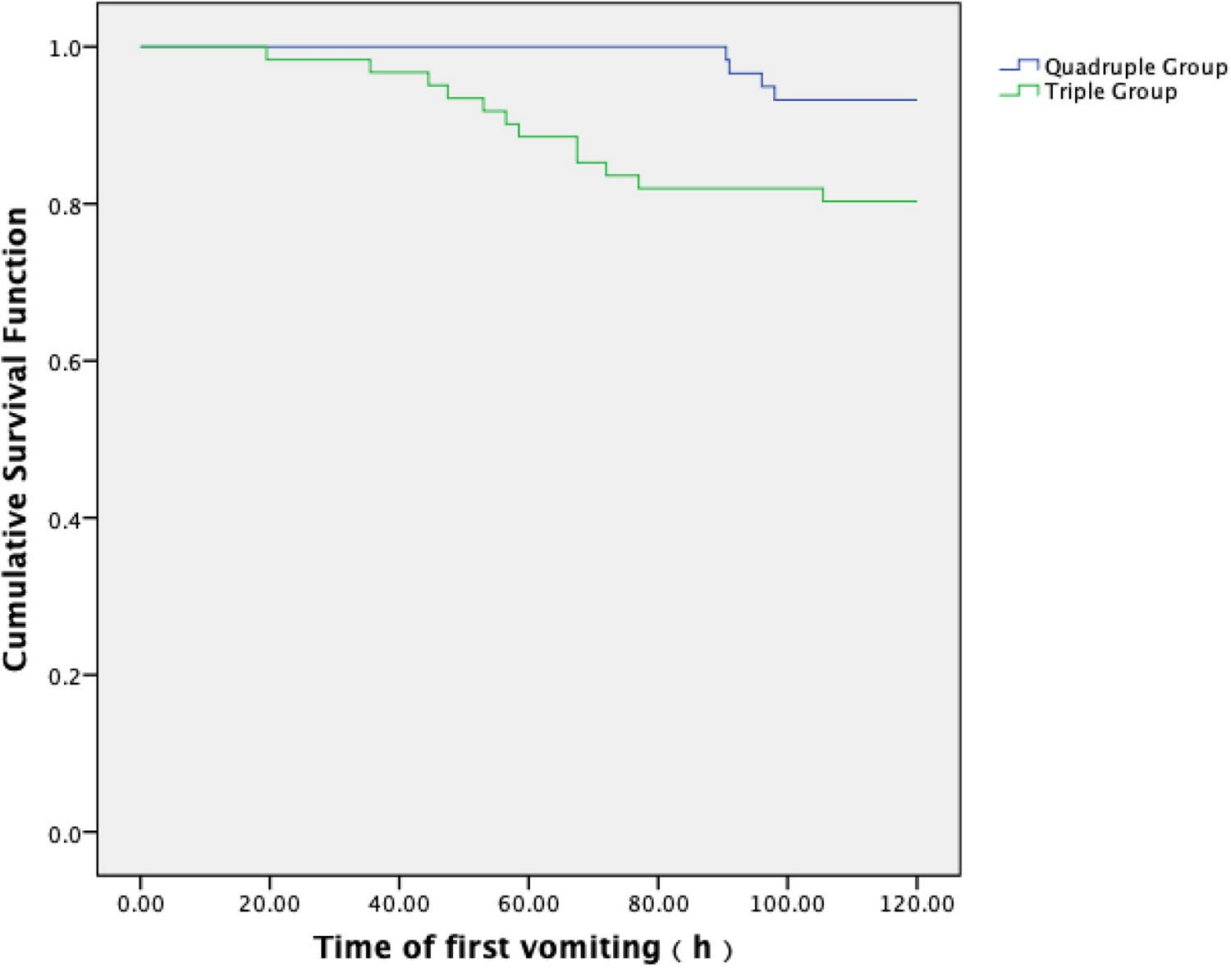
Comparison of time to first vomiting between two groups

Kaplan–Meier curves of time to first emesis were obviously longer in the quadruple regimen group than that in the triple regimen group (*P* = 0.031) (Fig. 2).

### Tolerability

The most common aprepitant- and olanzapine-related AEs of the total patients were recorded. AEs included sedation, fatigue, and constipation. The occurrences were observed in 57.6% (34/59), 54.2% (32/59), and 22.0% (13/59) of patients in the quadruple group vs 50.8% (31/61), 52.5% (32/61), and 13.1% (8/61) in the triple group (*P* = 0.454, *P* = 0.854, *P* = 0.199). The difference of AEs between two groups did not reach statistical significance. No grade 3 or 4 adverse events were observed in this study, and no patients interrupted the study because of undesired sedation.

## Discussion

Navari et al. conducted a phase III study revealing that olanzapine (10 mg) combined with NK-1RA, 5HT3-RA, and dexamethasone standard therapy was superior at overall phase after chemotherapy, both at the primary endpoint (no nausea) and the secondary endpoint (CR rate)[14]. Quadruple regimen therapy has been recommended antiemetic therapy for HEC in clinical guidelines of the Multinational Association of Supportive Care in Cancer/European Society for Medical Oncology (MASCC/ESMO), the American Society of Clinical Oncology (ASCO), and the National Comprehensive Cancer Network (NCCN)[16]. However, the majority of trials involved antiemetic research have investigated patients receiving single-day cisplatin chemotherapy, and multiple-day chemotherapy (MDC) is one of the most neglected and challenging areas of antiemetic research due to the overlap of acute phase and delayed phase[1].

So, it is meaningful for us to conduct this randomized, controlled, clinical trial of patients receiving 3-day cisplatin-based chemotherapy to compare the antiemetic effectiveness of three-drug and two-drug combinations plus olanzapine. The primary and secondary endpoints (CR rate) during OP and DP did not reach statistical significance, although there was higher CINV control rate for 5 mg olanzapine plus triple therapy with aprepitant, tropisetron, and dexamethasone in quadruple group compared with that of 5 mg olanzapine plus tropisetron and dexamethasone group, omitting aprepitant in triplet group during the early, later, and overall assessment phases. The primary endpoint did not reach statistical significance in this study which was inconsistent with previous reports by Navari et al.[14]. The possible explanations for those differences may be complex. On the one hand, we designed 5 mg olanzapine rather than NK-1 receptor antagonist-based triplet therapy as the control group because a phase 3 trial showed that olanzapine 10 mg was comparable to aprepitant in the control of CINV, but somnolence is a major side effect[6]. And olanzapine 5 mg has shown equivalent activity to olanzapine 10 mg and a favourable safety in relation to somnolence in phase II study; thus, it is reasonable for this clinical design[7]. On the other hand, we adopted CR as primary endpoint that was different from previous olanzapine combined triple therapy with NK-1RA, 5HT3-RA, and dexamethasone quadruple regimen clinical studies which adopted no nausea or TC as primary endpoint[14, 15]. As we know, nausea was better controlled by olanzapine in delayed period and overall period, so it may explained that the TC rates were identical in both groups in our study[6]. Besides these, a randomized, double-blind, placebo-controlled, phase III study enroled 710 patients to evaluate the efficacy of olanzapine 5 mg with triplet antiemetic therapy aprepitant, tropisetron, and dexamethasone adopted CR in the delayed phase as primary endpoint. The proportion of patients who achieved a complete response was 79% vs 66% (*P* < 0.0001)[8], and our clinical results of 76% vs 67% were highly in accordance with this phase III study, and a relative smaller sample size in this study than those in phase III trial may be one of the reasons of not reaching statistical significant in OP and DP[8, 14]. Furthermore, the different doses of 5-HT3RA, DXM, olanzapine, and given number of days in the study also affected the results [8, 14]. This study and J-FORCE both adopted 5 mg olanzapine achieved higher CR rate compared with a phase III study which adopted 10 mg olanzapine[8, 14]. The different time cut-off points between acute phase and delayed phase in multiple-day cisplatin-induced chemotherapy may affect the CR rate, as shown by H. F. Gao that CR declined about 20% when acute phase cut-off point switched from 24 to 72 h[17]. We defined 24 h as acute phase in this study, and 25% enroled patients received < 70 mg/m^2^ cisplatin in J-FORCE study may also explained the higher CR in some degree.

Whatever we got, such a result in this study of 5 mg olanzapine plus tropisetron and dexamethasone, omitting aprepitant compared with 5 mg olanzapine plus triple therapy with aprepitant, tropisetron, and dexamethasone, had identical control rate for prevention of CINV induced by cisplatin multiple-day chemotherapy.

According to FLIE, this study exhibited identical FLIE index of nausea or vomiting life quality in quadruple group compared to the triplet group. Kaplan–Meier curves of time to first emesis in the quadruple group were obviously longer than the triple group (*P* = 0.031), and this was in accordance with the secondary endpoint CR rate in AP, or no vomiting in OP was significantly higher in the quadruple group. This supports the superiority of control of chemotherapy-induced vomiting (CIV) by 5 mg olanzapine-based quadruple regimen therapy induced by cisplatin multiple-day chemotherapy.

The most common aprepitant- and olanzapine-related AEs included sedation, fatigue, and constipation. The occurrences between two groups were identical, and it was consistent with other studies examining aprepitant and olanzapine-related AEs[4, 8, 14]. In general, the tolerability in the study was safe, no grade 3 or 4 adverse events were observed in this study, and no patients discontinued the study because of undesired sedation.

## Conclusion

In summary, 5 mg olanzapine plus tropisetron and dexamethasone, omitting aprepitant therapy, may be an alternative regimen in prevention of CINV induced by multiple-day cisplatin chemotherapy. Larger sample sizes of clinical studies are ongoing to further confirm the advantage of aprepitant on the basis of 5 mg olanzapine combined with new generation of 5-HT3RA palonosetron and NK-1RA fosaprepitant for the prevention of CINV induced by multiple-day cisplatin chemotherapy.

## Data Availability

All data generated or analysed during this study are included in this published article (and its supplementary information files).
